# Aerotolerant Thiosulfate-Reducing Bacterium *Fusibacter* sp. Strain WBS Isolated from Littoral Bottom Sediments of the White Sea—Biochemical and Genome Analysis

**DOI:** 10.3390/microorganisms11071642

**Published:** 2023-06-23

**Authors:** Andrei L. Brioukhanov, Vitaly V. Kadnikov, Alexey V. Beletsky, Alexander S. Savvichev

**Affiliations:** 1 Faculty of Biology, Lomonosov Moscow State University, 119234 Moscow, Russia; 2 Skryabin Institute of Bioengineering, Research Center of Biotechnology, Russian Academy of Sciences, 117312 Moscow, Russia; vkadnikov@bk.ru (V.V.K.); mortu@yandex.ru (A.V.B.); 3 Winogradsky Institute of Microbiology, Research Center of Biotechnology, Russian Academy of Sciences, 117312 Moscow, Russia; savvichev@mail.ru

**Keywords:** *Fusibacter*, thiosulfate-reducing bacteria, antioxidant defense, genome sequencing, White Sea, marine bottom sediments

## Abstract

The strain WBS, an anaerobic, psychro- and halotolerant bacterium belonging to the genus *Fusibacter*, was isolated from the littoral bottom sediments of the White Sea, Arctic, Russia. *Fusibacter bizertensis* WBS grew at temperatures between 8 and 32 °C (optimum growth at 18–20 °C), pH between 5.2 and 8.3 (optimum growth at pH 7.2), and at NaCl concentrations between 0 and 70 g L^−1^ (optimum growth at 32 g L^−1^). It reduced sulfate, thiosulfate, and elemental sulfur into sulfide, and, probably, the strain is able to disproportionate thiosulfate. The strain also utilized a wide range of substrates as it is a chemoorganotrophic bacterium. Analysis of the sequenced genome revealed genes for all enzymes involved in the Embden–Meyerhof glycolytic pathway as well as genes for the non-oxidative stage of the pentose phosphate pathway. The presence of genes encoding aldehyde dehydrogenases and alcohol dehydrogenases also suggests that, in addition to acetate, alcohols can also be the fermentation products. The strain possessed superoxide dismutase and peroxidase activities and the ability to consume O_2_, which is in full accordance with the presence of corresponding genes of antioxidant defense in the genome. The phylogenetic analysis suggested that the strain WBS is the closest relative of *Fusibacter bizertensis* LTF Kr01^T^ (16S rRNA gene sequence similarity 98.78%). Based on biochemical and genomic characteristics, the strain WBS is proposed to represent a novel aero-, halo- and psychrotolerant strain from the genus *Fusibacter*, isolated for the first time among its members from cold oxygenated marine bottom sediments.

## 1. Introduction

The water areas of the Arctic Seas are the most vulnerable due to climate change and the growing pollution of the oceans. Therefore, increased attention is given to the environmental issues of the northern maritime regions. The research on bioresource potential of the Arctic Seas, which are particularly susceptible to anthropogenic impacts (i.e., various pollutants carried by the Atlantic waters, runoff from the large Siberian Rivers, as well as radioactive burials on the sea shelf), is of key importance. The construction of roads, dams, embankments, and tidal stations on the Arctic coast causes a decrease in natural water exchange, which leads to the appearance of anoxic bottom waters and bottom sediments [1]. Sulfate-reducing bacteria (SRBs) are the main component of the microbial communities in the marine water column and bottom sediments under conditions of oxygen deficiency.

Based on the results of a number of Arctic marine expeditions, it was found that the processes of sulfate reduction in the bottom sediments of the Arctic Seas could be very active [2,3]. Several strains of SRB isolated from such cold marine sediments into pure cultures were able to exist in an active state even at negative temperatures [4,5].

SRBs are obligate anaerobic microorganisms that exhibit dissimilatory in sulfate reduction. Many of them need not only the absence of oxygen in the environment but also a low redox potential for growth. However, many SRB species possess effective antioxidant enzymatic systems [6], which allow them to preserve metabolic activity even under unfavorable conditions of aerobiosis. It is well known that the main terminal electron acceptor for SRBs is sulfate (SO_4_^2−^), which is reduced to H_2_S during sulfate respiration. Many SRBs can also reduce sulfite (SO_3_^2−^), thiosulfate (S_2_O_3_^2−^) and, more rarely, elemental sulfur (S^0^). Electron donors are predominantly low-molecular organic compounds, which are formed during the anaerobic decomposition of biomass, as well as molecular hydrogen [7]. SRBs are widely distributed in various anaerobic ecosystems where sulfate is abundant, such as marine bottom sediments or sewage sludge. Sulfate reducers play an important role in the global cycles of sulfur and carbon, especially in the World Ocean [8,9], but in addition to them, thiosulfate- and sulfur-reducing microorganisms also have a significant influence on the sulfur biogeochemical cycle.

The genus *Fusibacter* belongs to the order *Clostridiales*, class *Clostridia*, and phylum *Firmicutes*. Members of this genus are chemo-organotrophic obligate anaerobic bacteria that are able to perform thiosulfate reduction. Only five species of the genus *Fusibacter* have been characterized thus far. They are *Fusibacter paucivorans*, isolated from reservoir water from an oil-producing well in Congo [10]; *Fusibacter tunisiensis*, isolated from sludge in an anaerobic reactor used to treat olive-mill wastewater in Tunisia [11]; *Fusibacter bizertensis*, isolated from a corroded kerosene storage tank in Tunisia [12]; *Fusibacter fontis*, isolated from a mesothermic Tunisian spring [13]; and *Fusibacter ferrireducens*, isolated from mangrove sediment sampled at the Jiulong river estuary in China [14]. While *F. paucivorans*, *F. tunisiensis*, *F. bizertensis*, and *F. ferrireducens* used both thiosulfate and elemental sulfur as electron acceptors (the last named species could also reduce sulfate), only elemental sulfur was reduced into sulfide by *F. fontis* [10,11,12,13,14].

Thus, the study of the metabolic activities of the sulfur cycle bacteria as well as their phylogenetic diversity in the world is still poorly explored but an extremely promising area of the World Ocean—the cold Arctic Seas—that can be explored using a complex combination of molecular, genetic, biochemical, and traditional microbiological methods, and is a very relevant and interesting scientific task. 

Here, we report on the isolation and characterization of a novel psychro- and halotolerant and sulfate-, thiosulfate-, and sulfur-reducing anaerobic bacterium strain WBS, belonging to the genus *Fusibacter* and isolated from bottom sediments in the littoral zone of the White Sea (Russia). It should be noted that due to the presence of water exchange with the mainland and waters of the Barents Sea, as well as the active mixing of waters because of tidal currents and anthropogenic influences, the coastal zones of the White Sea have unique hydrological and hydrochemical regimes in comparison with other Arctic Seas. In the White Sea bays, for example, the destruction of organic matter prevails over its formation. When studying the coastal zones of the White Sea, namely the Kandalaksha and Dvina bays, it was shown that the number of microorganisms, their productivity, and metabolic activity (especially, sulfate reduction, methane production, and oxidation) were much higher than in the open parts of the sea [15].

According to the results of the BLASTN analysis of a V3–V4 region (1482 bp) of the 16S rRNA gene, the isolated pure culture has the highest homology (98.78%) to *Fusibacter bizertensis*. Since the members of the *Fusibacter* genus had never been isolated before from marine bottom sediments (in particular, in the cold Arctic), the main aim of our research was to investigate the whole genome as well as to perform biochemical experiments to understand the metabolic capabilities of the strain WBS and its ability to survive in the oxygenated littoral sea zone.

## 2. Materials and Methods

### 2.1. Sampling

Samples were collected in August from the littoral bottom sediments of Kandalaksha Bay of the White Sea, near the Polar Circle (close to the Biological Station of Lomonosov Moscow State University, North Karelia, Russia). The samples were placed into sterile 50 mL Falcon tubes with hermetic screw caps, covered (2:1) with sterile buffer solution (50 mM Tris-HCl, 150 mM NaCl, 100 mM Na_2_EDTA, and pH 8.0) and kept at 4 °C before transporting to the laboratory. A modified Hungate technique, using vacuum degassing and argon flow into Hungate tubes with a 1 cm^3^ sample inside, was applied for the further isolation and cultivation of the obligate anaerobic microbial cultures [16].

### 2.2. Isolation and Cultivation of the Strain

The basal nutrient medium for marine SRBs [7] used for isolation contained (per L of distilled water) 4.0 g Na_2_SO_4_, 0.2 g KH_2_PO_4_, 0.25 g NH_4_Cl, 13.5 g NaCl, 0.4 g MgCl_2_ × 6H_2_O, 0.5 g KCl, 0.1 g CaCl_2_, 0.5 g yeast extract (Difco), 0.025 g FeSO_4_ × 7H_2_O, 1 mL resazurin, 1 mL Widdel trace element solution, and 1 mL Widdel vitamin solution [17]. The pH was adjusted to 7.5 with a 10 M KOH solution. Additionally, the nutrient medium was vacuum degassed under high purity argon flow, then dispensed into Hungate tubes, and subsequently sterilized by autoclaving at 111 °C for 20 min. Before inoculations, 0.1 mL 2 M sodium acetate, 0.1 mL 10% (*w*/*v*) NaHCO_3_, 0.1 mL 5% (*w*/*v*) Na_2_S, and 50 μL 50% (*w*/*v*) sodium lactate were injected by syringes from anaerobic sterile stock solutions into Hungate tubes containing 9 mL basal nutrient medium.

The cultures were purified by the repeated use of the Hungate roll-tube method [16] and nutrient medium that was solidified with 2.0% (*w*/*v*) agar before transfer of each isolated colony back into liquid nutrient medium for marine SRBs. At first, to obtain enrichment cultures, 1 cm^3^ of the bottom sediment sample was placed into a Hungate tube containing 9 mL basal nutrient medium with lactate as an energy source. This procedure was repeated to obtain serial dilutions (from 10^−1^ to 10^−10^) and the inoculated Hungate tubes were then incubated at 20 °C for 5–20 days. To obtain the pure cultures from the corresponding dilution, enrichment cultures were reinoculated several times under the same growth conditions prior to the isolation of separate colonies. For isolation, the cultures were serially diluted 10-fold with agar basal nutrient medium in roll tubes sealed with a gas-tight butyl rubber stopper. Several black colonies that appeared were picked separately in sterile conditions; they were about 2 mm in diameter after 5–7 days of incubation. One of the cultures, most closely related to *F. bizertensis* according to the V3–V4 region sequence of the 16S rRNA gene, was chosen for further characterization and designated as strain WBS (White Sea Biological Station).

Microbiological experiments were performed in triplicates using Hungate tubes containing 9 mL of the basal nutrient medium and 10% of fresh inoculum. Temperature, pH, and NaCl concentration ranges (5–50 °C, pH 4.5–9.5, 0–100 g NaCl L^−1^, correspondingly) for strain growth were defined using the basal nutrient medium supplemented with 22 mM lactate or d-glucose. The pH of the basal nutrient medium, prepared on 200 mM sodium phosphate buffer solution with the corresponding pH instead of water, was adjusted with sterile stock solutions of 1 M HCl or 10% (*w*/*v*) Na_2_CO_3_ after inoculation. NaCl was weighed directly in the Hungate tubes prior to the addition of the nutrient medium. The isolate was subcultured twice under the same experimental conditions prior to the determination of its growth in order to minimize the lag phase due to the change in cultivation conditions.

For substrate utilization experiments, the basal nutrient medium was used. The substrates (lactate, d-arabinose, d-fructose, d-galactose, d-glucose, d-mannitol, d-ribose, d-sucrose, d-xylose, d-trehalose, d-mannose, d-sorbose, cellobiose, maltose, and propionate) were injected from sterile stock solutions into Hungate tubes to reach a final concentration of 22 mM. To test for electron acceptors, 28 mM sodium sulfate, 20 mM sodium thiosulfate, or 2% (*w*/*v*) elemental sulfur were added to the nutrient medium supplemented with lactate or d-glucose. 

The morphology of cells from the exponential phase and purity of the cultures during growth experiments were determined by light microscopy at 1000× magnification using fuchsine staining. Gram staining was also performed on heat-fixed cells using the Gram Stain Kit (BD, Franklin Lakes, NJ, USA). Furthermore, growth of the cultures was measured spectrophotometrically at λ = 575 nm, and sulfide was determined colorimetrically with N,N-dimethyl-*p*-phenylenediamine [18] on a SPEKOL 21 semiautomatic spectrophotometer (Analytic Jena, Jena, Germany) at λ = 670 nm.

### 2.3. Determination of Specific Activities of Antioxidant Enzymes

Cell pellets obtained after centrifugation of the 200 mL cultures from the exponential phase of growth (4000× *g*, 30 min, 4 °C) were resuspended in 10 mL of ice-cold 50 mM potassium phosphate buffer solution (pH 7.8). The cells were disrupted using an ultrasonic disintegrator UZDN-2T, Russia (40 μA, 22 kHz, 10 times for 30 s with 1 min pauses for cooling in ice). Subsequently, cell debris was removed by centrifugation (28,000× *g*, 30 min, 4 °C) and cell-free extracts were used for immediate determination of enzymatic activities.

Superoxide dismutase (SOD) activity was determined spectrophotometrically at λ = 550 nm by the xanthine oxidase–cytochrome *c* method [19]. One unit of SOD is defined as the amount of enzyme required to inhibit the reduction rate of cytochrome *c* by 50% in a coupled system using xanthine and xanthine oxidase (Sigma-Aldrich, St. Louis, MO, USA) at a pH of 7.8 and 25 °C. 

Catalase activity was determined spectrophotometrically at a pH of 7.0 and 25 °C by monitoring the decrease in absorbance of 13 mM H_2_O_2_ at λ = 240 nm (ε_240_ = 39.4 M^−1^ cm^−1^). Additionally, the total peroxidase activity was determined spectrophotometrically using 2,2′-azino-bis(3-ethylbenzthiazoline-6-sulfonic acid) as a substrate [20]. The oxidation of ABTS (Sigma-Aldrich, St. Louis, MO, USA) by H_2_O_2_ in the peroxidase reaction system was recorded by a linear increase in absorbance at λ = 405 nm (ε_405_ = 36.8 M^−1^ cm^−1^). One unit of peroxidase activity is defined as the amount of enzyme required to oxidize 1 μmole of ABTS per min at a pH of 5.0 and 25 °C. An NADH-dependent peroxidase activity assay was also performed, as described by Coulter et al. [21].

The oxygen consumption activity of the whole cells was measured polarographically with a Clark-type electrode in 0.1 M Tris-HCl and 0.15 M NaCl buffer solution (pH 7.5) at 25 °C, with menadiol (50 µM) or NADH (3 mM) as an electron donor. The chemical reaction was subtracted to obtain the enzymatic activity.

Finally, protein concentrations were determined by the Bradford method using the Protein Assay Kit from Bio-Rad Laboratories (USA) and bovine serum albumin as a standard.

All measurements of enzymatic activities were performed in triplicates and analyzed using the SigmaStat v.4.0 program (Systat Software, Chicago, IL, USA). 

### 2.4. Genome Analysis

Genomic DNA from bacterial culture was isolated using the Genomic DNA Purification Kit (Thermo Fisher Scientific, Waltham, MA, USA) according to the manufacturer’s protocol. Subsequently, the shotgun genome library was prepared using the NEBNext Ultra II DNA Library Prep Kit (New England BioLabs, Ipswich, MA, USA). The sequencing of this library was performed on an Illumina NovaSeq 6000 System (Illumina, San Diego, CA, USA). Overlapping paired-end reads were merged using the FLASH v.1.2.11 software [22], and low-quality bases were trimmed using the Sickle v.1.33 software [https://github.com/najoshi/sickle (accessed on 14 December 2022)]. The Illumina reads were de novo assembled into contigs using the SPAdes v.3.15.4 software [22]. Gene search and annotation of the genome were then performed using the NCBI Prokaryotic Genome Annotation Pipeline [23] or RAST server v.2.0 [24], followed by a manual correction of the annotation by comparing the predicted protein sequences with the U.S. National Center for Biotechnology Information (NCBI) databases. The average amino acid identity (AAI) and average nucleotide identity (ANI) between the selected whole genomes were calculated using appropriate scripts from the Enveomics Collection [25]. Lastly, the G + C content of the genomic DNA was determined using the whole genome sequence. 

## 3. Results

### 3.1. Morphological and Physiological Properties of the Strain WBS

Cultures of the strain WBS contained slightly motile, Gram-positive staining, long rod-shaped cells. They appeared singly or in pairs, and spores were not observed. The colonies seen in the roll tubes were round, about 2 mm in diameter, and of black color after 5–7 days of incubation at 20 °C. 

The strain WBS is anaerobic and grew extremely slow in oxidized medium (indicated by the pink color of resazurin), but cells remained viable under up to 4% (*v*/*v*) O_2_ flow, so the strain is aerotolerant. The strain is also psychrotolerant, growing from 8 °C to 32 °C with optimum growth at temperatures between 18 and 20 °C. It grew at sodium chloride concentrations ranging from 0 to 70 g NaCl L^−1^, with optimum growth at 32 g NaCl L^−1^ (halotolerant strain, can be considered as slightly halophilic). Growth occurred between pH 5.2 and 8.3, with the optimum pH for growth being 7.2 (neutrophilic strain).

Additionally, the strain WBS grew very well with the following substrates as electron donors and energy sources: lactate, d-glucose, d-fructose, d-ribose, and d-galactose. Cultures grew weakly with significantly decreased growth rate and biomass yield with d-mannitol, d-xylose, d-mannose, maltose, and cellobiose as substrates (Table 1). The strain did not utilize d-arabinose, d-sucrose, d-sorbose, d-trehalose, and propionate. Moreover, the strain WBS used sulfate, thiosulfate and elemental sulfur as electron acceptors, produced during growth on d-glucose up to 3.9, 13.4, and 6.1 mM H_2_S, correspondingly. The best growth was achieved on thiosulfate.

### 3.2. Phylogenetic and Genome Analysis of the Strain WBS

Phylogenetic BLAST analysis of the 16S rRNA gene sequence containing a 1482 bp V3–V4 fragment revealed that the strain WBS represents a novel strain of the *Fusibacter* genus, which belongs to family XII of the order *Clostridiales*. Strain WBS was most closely related (98.78% similarity of 16S rRNA gene sequence) to the *F. bizertensis* strain LTF Kr01^T^, isolated from a corroded kerosene storage tank [12]. It should be noted that the *Fusibacter* genus is quite rare (five species are described up to date—*F. paucivorans*, *F. tunisiensis*, *F. bizertensis*, *F. fontis*, and *F. ferrireducens*), and its members have never been isolated from sea bottom sediments before.

**Figure 1:**
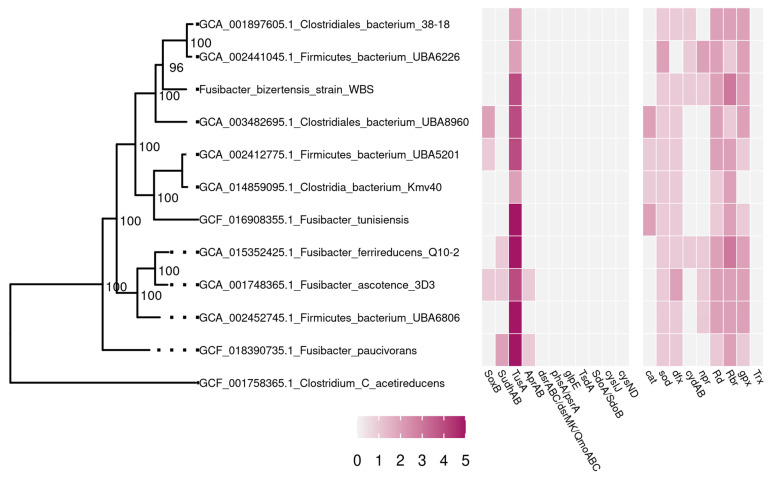
Phylogeny of bacteria of the genus *Fusibacter* based on the analysis of concatenated sequences of conserved marker genes. The *Clostridium acetireducens* genome was used as an outgroup. Branch support levels are determined using a Bayesian test in PhyML. Gene encoding is based on the following: *soxB*—thiosulfohydrolase; *sudhAB*—sulfide dehydrogenase; *tusA*—sulfur relay protein TusA; *aprAB*—adenylylsulfate reductase; *dsrAB*—dissimilatory sulfite reductase; *dsrMK*—sulfite reduction-associated electron transfer complex proteins, DsrM and DsrK; *qmoABC*—APS reductase-associated electron transfer complex; *phsA*/*psrA*—polysulfide reductase/thiosulfate reductase chain; *glpE*—thiosulfate sulfurtransferase; *tsdA*—thiosulfate dehydrogenase; *sdoA*/*sdoB*—sulfur dioxygenases; *cysIJ*—sulfite reductase (NADPH) flavoprotein; *cysND*—sulfate adenylyltransferase; *cat*—catalase; *sod*—superoxide dismutase; *dfx*—desulfoferrodoxin; *cydAB*—cytochrome *d* ubiquinol oxidase; *npr*—NADH peroxidase; *rd*—rubredoxin; *rbr*—rubrerythrin; *gpx*—glutathione peroxidase; *trx*—thiol peroxidase.

The genome of the strain WBS (the first sequenced genome of *F. bizertensis* species) was obtained in the form of 137 contigs with a total length of 3,806,551 bp. The DNA G + C content of the strain WBS was 35.1 mol%. According to the CheckM service, out of 247 conserved single-copy genes, this genome contains 235 genes in one copy, 10 in two copies, and 1 in three copies, which indicates 99.29% completeness of the genome. As a result of the genome analysis, 5S, 16S, and 23S rRNA genes were found, as well as 74 tRNA genes. Based on the results of genome annotation, 3569 potential protein-coding genes were predicted, of which the physiological functions of only 1931 can be predicted by comparison with the NCBI databases. To analyze the phylogeny of the *Fusibacter* sp. strain WBS, a phylogenetic tree was constructed based on the concatenated sequences of 43 conserved marker genes (Figure 1). 

The analysis of the similarity of nucleotide sequences of full genomes [26] showed that ANI between genomes is 76–77% and AAI is 61–69%, which indicate that the strain belongs to the same genus *Fusibacter* (Table S1).

### 3.3. Metabolic Capabilities of the Strain WBS According to Genome Analysis

Genome analysis revealed genes for all enzymes of the Embden–Meyerhof glycolytic pathway as well as genes for the non-oxidative stage of the pentose phosphate pathway. There are no known ways of autotrophic carbon fixation in the genome. As a reserve polysaccharide strain, WBS can use glycogen, as evidenced by the presence of genes encoding key enzymes involved in its synthesis and degradation—glucose-1-phosphate adenylate transferase, glycogen synthase, 1,4-alpha-glucan coupling enzyme, and glycogen phosphorylase.

Pyruvate, formed as a result of glycolysis, can be converted to acetyl-CoA by pyruvate–ferredoxin oxidoreductase. The oxidation of acetyl-CoA to form acetate and generate ATP can be carried out in a two-step reaction by phosphate acetyltransferase and acetate kinase, while no acetyl-CoA synthetase has been found. The presence of aldehyde and alcohol dehydrogenases suggests that, in addition to acetate, alcohols can also be the products of carbohydrate fermentation. Acetyl-CoA formed from pyruvate can enter the tricarboxylic acid cycle. However, this cycle is incomplete and is probably only used for biosynthetic purposes. The reduced products formed in the fermentation pathways, NADH and reduced ferredoxin, can be re-oxidized by [Fe-Fe]-type hydrogenases [27].

The strain WBS has several mechanisms for generating a transmembrane ion gradient, which can be used by the membrane-bound ATP synthase F_0_F_1_ type for ATP synthesis. First, there is a complete set of genes encoding the membrane-bound ion-transporting Rnf complex, which can function either in an anabolic way, reducing ferredoxin and oxidizing NADH, or in a catabolic way, oxidizing ferredoxin, reducing NAD^+^, and transporting protons or sodium ions across the cytoplasmic membrane [28].

Based on the gene order, *rnfCDGEAB*, the Rnf operon of the strain belongs to type 3, commonly found in the *Firmicutes* phylum, where it acts catabolically as an energy-storing ferredoxin: NAD^+^ oxidoreductase. Two other identified enzymes can also contribute to the generation of a transmembrane ion gradient, V-type ATPase, which uses ATP hydrolysis to transport protons, and membrane-bound pyrophosphatase, which uses pyrophosphate hydrolysis to translocate protons or Na^+^ ions. The absence of lysine in the signature sequence GNXX (K/A) [29] indicates that this pyrophosphatase transports sodium ions rather than protons. H^+^/Na^+^ antiporters encoded in the genome can provide the balance between H^+^ and Na^+^ concentrations.

The major genes for dissimilatory sulfate reduction encoding dissimilatory sulfite reductase (*dsrAB*) were not found in the genomes of *Fusibacter*, considered in the literature as thiosulfate-reducing bacteria [10,11,12]. The genes for polysulfide reductase/thiosulfate reductase (*phsA*/*psrA*), thiosulfate sulfurtransferase (*glpE*), thiosulfate dehydrogenase (*tsdA*), sulfur dioxygenases (*sdoAB*), and sulfate adenylyltransferase (*cysND*) were also not detected. However, the genes encoding sulfide dehydrogenase subunits (*sudhAB*), which catalyze the reduction of polysulfide to H_2_S with NADPH as the electron donor, were found in three *Fusibacter* genomes (Figure 1).

### 3.4. Systems of Antioxidant Defense of the Strain WBS 

Although SRBs are considered as strict anaerobic microorganisms, many species are relatively aerotolerant and have been found at oxic–anoxic interfaces and in biotopes periodically exposed to oxygen [30]. Therefore, many SRBs have different enzymatic systems of protecting themselves from oxidative stresses, including classical enzymes of reactive oxygen species (ROS) detoxification (superoxide dismutase, catalase, peroxidases) and unique alternative enzymes with superoxide reductase (desulfoferrodoxin, neelaredoxin) and NADH-dependent peroxidase (rubrerythrin, nigerythrin) activities [6].

Analysis of the *Fusibacter* genomes available in the GeneBank database revealed genes involved in oxidative stress response in cells of these anaerobic bacteria. Genes encoding superoxide dismutase (*sod*), superoxide reductase (*dfx*), rubredoxin (*rd*), and rubrerythrin (*rbr*) were found in all genomes, which protect a cell from the produced ROS. In addition, the catalase-encoding gene (*cat*) was found in four of the eleven *Fusibacter* genomes and the gene for glutathione peroxidase (*gpx*) was detected in almost all of them (Figure 1).

In full correspondence with genomic data, *Fusibacter* exhibited relatively high superoxide dismutase (SOD) (17.4 U mg^−1^ protein) and moderate peroxidase (3.2 U mg^−1^ protein) activities, but no catalase activity was observed in cell-free extracts (Table 1). The presence of NADH-dependent peroxidase activity corresponds well with the detection of the *rbr* gene encoding rubrerythrin. Sequence analysis of the *dfx* gene revealed that *Fusibacter* spp. desulfoferrodoxin was homologous to the two iron-containing superoxide reductases (SORs) common for SRBs. Probably, in the *Fusibacter* genus, SOD and SOR (desulfoferrodoxin) play a major role in protecting cells from ROS, contributing to the relative aerotolerance of these bacteria. Moreover, interestingly, based on the detection of *cydAB* genes encoding subunits I and II of cytochrome d ubiquinol oxidase, the washed cells of the *Fusibacter* sp. Strain WBS exhibited relatively high O_2_ reduction activity (11 ± 2 nmol O_2_ min^−1^ mg^−1^ protein), which was dependent of menadiol, an analog of menaquinols which are found in anaerobic bacteria. 

## 4. Discussion

Phylogenetic analysis of the amplified V3–V4 region (1482 bp) of the 16S rRNA gene indicated that the strain WBS is a member of the *Fusibacter* genus, which belongs to family XII of the *Clostridiales* order and the *Firmicutes* (*Bacillota*) phylum. The closest relative was found to be the *F. bizertensis* strain LTF Kr01^T^ (98.78% similarity of 16S rRNA gene sequence), which was isolated from a corroded kerosene storage tank in Tunisia [12]. The ecological niche for this strain completely differs from the Arctic marine bottom sediments. In the genome of the strain WBS, no genes responsible for hydrocarbon degradation were found. Unlike *F. bizertensis* LTF Kr01^T^ that grew at 0.5% (*w*/*v)* optimal NaCl concentration [12], the strain WBS was much more tolerant to the presence of NaCl in the nutrient medium, being slightly halophilic, with optimal growth at 3.2% (*w*/*v*) NaCl. Furthermore, the strain WBS grew at low temperatures from 8 °C with optimal growth at 18–20 °C (Table 1). All other known members of the *Fusibacter* genus [10,11,12,13] are not psychrotolerant but are classified as mesophilic bacteria (grew from 15–20 °C with optimal growth at 30–37 °C). 

The strain WBS, isolated from oxygenated littoral sediments, is an aerotolerant (can even be considered as microaerophile), anaerobic bacterium, remaining viable under 4% (*v*/*v*) O_2_ flow and possessing key genes for antioxidative defense and relatively high specific activities of corresponding enzymes such as SOD and peroxidases. *F. bizertensis* LTF Kr01^T^ tolerated up to 2% O_2_ [12], whereas *F. tunisiensis* exhibited tolerance just up to 1% O_2_ [11]. The genome analysis for genes encoding key antioxidant enzymes as well as the measurements of corresponding enzymatic activities were performed for the first time on *Fusibacter* spp. cells. Genomic features, which contribute to adaptations of *Fusibacter* members to oxygenated environments, include several genes encoding enzymes with the capacity to remove ROS—superoxide dismutase (*sod*) and desulfoferrodoxin (*dfx*) as a superoxide reductase provide the defense against superoxide radicals, whereas rubrerythrin (*rbr*) and glutathione peroxidase (*gpx*) reduce peroxides. In addition, some strains such as *F. ferrireducens* Q10-2^T^ and *F. bizertensis* WBS also possess *npr* and *cydAB* genes, encoding NADPH peroxidase and cytochrome *d* ubiquinol oxidase, correspondingly. The latter enzyme, when relating to aerotolerant anaerobes, is involved in oxygen consumption (but not respiration) as part of their complex antioxidant systems [6].

In comparison with the *F. bizertensis* strain, LTF Kr01^T^ [12], the strain WBS utilized quite similar range of substrates, wider than described for *F. paucivorans* [10]. Our analysis revealed genes encoding all enzymes involved in the Embden–Meyerhof glycolytic pathway in the genome of the strain WBS, as well as genes for the non-oxidative stage of the pentose phosphate pathway, and for the Leloir pathway of d-galactose catabolism. The strain WBS can also perform phosphoribosyl phosphate biosynthesis, and possesses genes encoding glucose/mannose, cellobiose, d-xylose, and maltose/maltodextrin transport systems. Interestingly, the strain WBS grew on lactate as an electron donor, whereas no other described members of the *Fusibacter* genus could use lactate, except the *Fusibacter* sp. 3D3 isolated from a hypersaline salt flat in Chile [31]. The end products of carbohydrate fermentation of *F. paucivorans* [10], *F. tunisiensis* [11], and *F. bizertensis* LTF Kr01^T^ [12] were acetate, CO_2_, and H_2_ (plus butyrate for *F. paucivorans*), but genome analysis of the strain WBS revealed the presence of genes encoding aldehyde and alcohol dehydrogenases. This fact suggests that, in addition to acetate, alcohols such as ethanol can also be produced from glucose metabolism by the strain WBS, which makes it similar, in this respect, to *F. fontis* [13] and *F. ferrireducens* [14]. 

It should also be surely noted that, similar to *F. paucivorans* SEBR 4211^T^ [10], *F. tunisiensis* BELH1^T^ [11], and *F. bizertensis* LTF Kr01^T^ [12], the strain WBS reduces thiosulfate to sulfide. Thiosulfate reduction is a common physiological process exhibited by some fermentative bacteria, such as *Thermoanaerobacter* spp. from oilfield ecosystems [32,33], which is thought to increase microbial-influenced corrosion processes [34]. It was shown using *F. paucivorans* that the use of thiosulfate as an electron acceptor causes a shift in the flow of electrons from butyrate to acetate, favoring acetate and H_2_S production [10]. In contrast to other members of the *Fusibacter* genus [10,11,12], with the exception of *F. ferrireducens* [14], which use thiosulfate and elemental sulfur only, the strain WBS could also use sulfate as a terminal electron acceptor. 

Surprisingly, our genome analysis revealed that the major genes for dissimilatory sulfate reduction, including dissimilatory sulfite reductase genes (*dsrAB*), were not found in the known genomes of *Fusibacter*; nevertheless, *F. ascotence* and *F. paucivorans* possess *aprAB* genes encoding adenylylsulfate reductase. However, the genes encoding sulfide dehydrogenase (*sudhAB*), which catalyzes the reduction of polysulfide to H_2_S with NADPH as the electron donor, were revealed in three *Fusibacter* genomes (Figure 1). It is not improbable that the members of the *Fusibacter* genus can perform disproportionation of thiosulfate and sulfite, as it was shown for *Desulfovibrio sulfodismutans* [35], *Desulfocapsa thiozymogenes* [36], and bacteria belonging to *Nitrospirota* [37]. Additionally, the hyperthermophilic obligate anaerobic bacterium *Thermotoga maritima* is able to utilize diverse carbohydrates, but the cellular mechanism of sulfate reduction for this species has not been fully understood yet; this strain also possesses NAD(P)H-dependent polysulfide reductase with S^0^ and thiosulfate reductase activities [38].

The DNA G + C content of the strain WBS was 35.1 mol%, which was lower than that of other members of the *Fusibacter* genus (37.4–43.0 mol%) [10,11,12,13,14,31]. The genome of the strain WBS (the first sequenced genome of the *F. bizertensis* species) consisted of 137 contigs with a total length of 3.81 Mbp (3569 potential protein-coding genes were predicted), whereas the first published draft genome sequence of a strain belonging to the *Fusibacter* genus was larger (5.11 Mbp) and arranged in 57 contigs (4700 predicted genes coded for proteins) [31].

The obtained physiological and genomic data clearly showed that the aero-, halo-, and psychrotolerant strain WBS, which is able to reduce inorganic sulfur compounds, represents a novel strain within the *Fusibacter* genus, which was isolated for the first time among its members from marine bottom sediments, in particular from the cold Arctic environment. Overall, this fact suggests that *Fusibacter* species may inhabit a broad range of different ecosystems and play a certain role in the biogeochemical sulfur cycle.

## Figures and Tables

**Table 1:** Physiological characteristics of *Fusibacter* sp. strain WBS in comparison with *F. bizertensis* strain LTF Kr01^T^ (latter according to [12]).

Characteristic (Optimum)	Strain WBS	Strain LTF Kr01^T^ [12]
Morphology	Long rods	Short rods
DNA G + C content, mol%	35.1	37.4
Temperature growth range	8–32 °C (18 °C)	15–40 °C (30 °C)
pH growth range	5.20–8.30 (7.20)	5.50–8.16 (7.20)
NaCl growth range	0–70 g L^−1^ (32)	0–50 g L^−1^ (5)
Electron acceptors		
Sulfate	+	−
Thiosulfate	+	+
Sulfur	+	+
Substrates utilized		
Lactate	+	−
d-Glucose	+	+
d-Fructose	+	+
d-Ribose	+	+
Cellobiose	±	+
d-Mannitol	±	+
d-Xylose	±	+
d-Galactose	+	+
d-Arabinose	−	−
Maltose	±	+
d-Sucrose	−	+
d-Trehalose	−	+
d-Mannose	±	−
d-Sorbose	−	−
Propionate	−	−
SOD activity	17.4 ± 1.5 U mg^−1^ prot.	
Catalase activity	−	
Peroxidase activity	3.2 ± 0.2 U mg^−1^ prot.	
NADH peroxidase activity	+	
O_2_-consumption rate	11 ± 2 nmol O_2_ min^−1^ mg^−1^ prot.	

+, Positive; −, negative; ±, positive but growth was weak.

## Data Availability

The whole-genome assembly of the *Fusibacter bizertensis* strain WBS has been deposited in DDBJ/ENA/GenBank under the BioProject accession number PRJNA957129.

## Supplementary Materials

The following supporting information can be downloaded at https://www.mdpi.com/article/10.3390/microorganisms11071642/s1. Table S1: The analysis of the average nucleotide identity (ANI) and average amino acid identity (AAI) of the whole genomes within *Fusibacter* genus.
